# Human Endothelial Cells Modulate CD4^+^ T Cell Populations and Enhance Regulatory T Cell Suppressive Capacity

**DOI:** 10.3389/fimmu.2018.00565

**Published:** 2018-03-23

**Authors:** Wen Chean Lim, Michael Olding, Eugene Healy, Timothy M. Millar

**Affiliations:** ^1^Dermatopharmacology, Clinical and Experimental Sciences, Faculty of Medicine, University of Southampton, Southampton, United Kingdom; ^2^Dermatology, University Hospital Southampton NHS Foundation Trust, Southampton, United Kingdom

**Keywords:** regulatory T cells, endothelial cells, immune regulation, cell–cell interaction, CD4^+^ T cells

## Abstract

Endothelial cells (ECs) line the luminal surface of blood vessels and have an active role in the recruitment of leukocytes, including immune cell activation. Regulatory T cells (Tregs) are immune suppressor cells that maintain peripheral tolerance and must interact with the endothelium as they traffic into tissue. We hypothesized that human ECs could modulate Tregs and their suppressor function. Cocultures of CD4^+^ T cells with human umbilical vein ECs (HUVECs) or dermal microvascular ECs (HDMECs) were conducted and analyzed for activation and proliferation after 72 and 120 h using flow cytometry. In monocyte-depleted cultures, human ECs were found to support CD4^+^ T cell proliferation in the presence of external mitogens phytohemagglutinin or anti-CD3/28 antibodies (aCD3/28). Activation was shown by CD25 expression in these cells that also transiently expressed the Treg transcription factor FOXP3. HUVECs supported the specific concurrent proliferation of both effector T cells and Tregs when cocultured with aCD3/28. Purified Tregs were also functionally activated by prior coculture with EC to suppress effector T (Teff) cell proliferation. Both direct coculture and indirect coculture of EC and Treg showed activation of the Treg suppressive phenotype. However, whereas HUVEC showed enhancement of suppression by both mechanisms, HDMEC only supported Treg suppressive activity *via* the contact-independent mechanism. In the contact-independent cultures, the soluble mediators IL-6, GM-CSF, or G-CSF released from ECs following interferon-γ activation were not responsible for the enhanced Treg suppressor function. Following direct coculture, Treg expression of inhibitory receptors PD-1 and OX40 was elevated while activated EC expressed the counter ligands programmed death ligand (PD-L)1 and PD-L2. Therefore, human ECs have a role in supporting T cell proliferation and increasing Treg suppressor function. This ability of EC to enhance Treg function could offer novel targets to boost Treg activity during inflammatory disorders.

## Introduction

The endothelium is a biologically active cellular site lining the luminal surface of blood vessels. Endothelial cells (ECs) contribute to the many physiological roles of the vasculature including coagulation, forming a selective barrier to regulate vessel permeability, and controlling vascular tone. In addition to these functions, ECs also have important immunological roles, in particular the control of leukocyte recruitment and activation (1). ECs express a variety of adhesion molecules, including selectins and integrins, which allow them to regulate recruitment and trafficking of leukocytes into an inflammatory site (2). ECs can also express surface molecules that mediate antigen presentation and provide costimulatory signals, including class I and class II major histocompatibility complexes and costimulatory molecules such as inducible T cell costimulator (ICOS)-ligand, programmed death ligand (PD-L) -1 and -2, OX40-ligand and 4-1BB-ligand (1). Evidence for the involvement of human ECs in regulating T cell activation include cytokine production and proliferation of T cells mediated *via* ICOS-L expression on human ECs and costimulation of resting memory CD4^+^ T cells to produce T helper (Th) -1 and Th2 cytokines (3). Conversely, inhibitory signals mediated through PD-L1 expression on human umbilical vein endothelial cells (HUVECs) have been shown to negatively regulate IL-2 and interferon (IFN)-γ production of phytohemagglutinin (PHA)-stimulated T cells (4).

Endothelial cells also have a role in the recruitment of regulatory T cells (Tregs). These cells were initially characterized by Sakaguchi et al. (5) as IL-2 receptor α-chain (CD25) expressing CD4^+^ T cells and subsequently the transcription factor Foxhead Box P3 (Foxp3) was shown to be necessary for Treg development and function (6, 7). Treg function is regulated by multiple mechanisms, including direct interaction with cells *via* costimulatory signals through PD-1 and OX40 and their corresponding ligands (8, 9) and indirectly by cytokine signaling *via* IL-6 and IL-10 (10, 11). Treg recruitment and migration into lymph nodes and peripheral tissue is pivotal in regulating their role in peripheral tolerance (12). In mice, Krupnick et al. (13) demonstrated that ECs derived from the thoracic aorta could selectively expand CD4^+^CD25^+^Foxp3^+^ Tregs in cocultures with CD4^+^ T cells. Later, Bedke et al. (14) showed that activated murine lung ECs increased the capacity of CD4^+^CD25^+^ Tregs to suppress effector T cell proliferation. More recently, human dermal ECs have been shown to induce expansion of Tregs and proinflammatory Th17 populations in cocultures with CD4^+^ T cells (15) but did not investigate the suppressive function of Tregs following endothelial interaction. While a further study showed that in rapamycin-treated HUVECs Treg suppressive activity was increased potentially through increased PD-L1 and PD-L2 expression (16).

Endothelial cells have, therefore, been proposed to induce Treg expansion and enhance Treg suppressive capacities but the evidence in humans and chronic inflammatory models is limited. We hypothesized that under chronic inflammatory cytokine activation the endothelium could potentially modulate T cell function in a manner that relates to chronic diseases of the skin. This aim of this present study was to demonstrate the capabilities of cytokine stimulated human ECs to modulate T cell differentiation and Treg function. This paper uses *in vitro* EC-T cell cocultures and shows that EC–Treg interactions are important for Treg activation and that differences exist between ECs of different lineages. We also show that ECs are capable of the induction and expansion of Tregs and that the potential mechanism(s) by which this occurs involves both direct contact and indirect signals to enhance the suppressive activity of Tregs. In the light of these and previous findings, the endothelium has a potential role to play in controlling chronic inflammation *via* both Teff and Treg activation and presents itself as a potential target for immune modulation in inflammation, cancer and autoimmune disease.

## Materials and Methods

### Reagent and Antibodies

PerCP-Cy5.5 conjugated anti-CD4 (RPA-T4) mAb, eFluor 450 conjugated anti-CD127 (eBioRDR5) mAb, and APC conjugated anti-FOXP3 (PCH101) mAb (eBioscience, UK). PE conjugated anti-CD25 (CD25-3G10) mAb (Life Technologies, UK). PE-Cy7 conjugated PD-1 (EH12.2H7) mAb and Brilliant Violet 421 conjugated OX40 (Ber-ACT35) mAb (Biolegend, UK). IFN-γ (human, leukocyte-derived) and tumor necrosis factor (TNF)-α (human, rDNA derived) (The National Institute for Biological Standards and Control, NIBSC, UK).

### Primary Human EC Culture

M199 media (Life Technologies, UK) with 20% pooled off the clot human serum (TCS Bioscience, UK), 100 U/mL penicillin, 100 µg/mL streptomycin, and 292 μg/mL l-glutamine (Life Technologies, UK) was used for HUVEC culture, whereas EC Growth Medium MV2 (Promocell; Heidelberg, Germany) was used for HDMEC culture. Porcine gelatin (0.2%, Sigma Aldrich, UK) was used as the coating matrix for EC culture.

Methods for isolating HUVECs were adapted from protocols published by Jaffe et al. (17) and Gimbrone et al. (18). Briefly, umbilical cords were collected from the Princess Anne Hospital, Southampton, UK from non-complicated natural vaginal births following agreed ethical collection protocols [Local Research Ethical Committee (LREC); Ref: 07/H0502/83]. Veins were cannulated and incubated with collagenase (1 mg/mL of type 1 collagenase ~250 U/mg, Worthington Biochemical; Lakewood, US) for 10 min in a 37°C water bath. HUVECs were collected by centrifugation and plated on to gelatin coated plastics and grown to confluence. HUVECs were used at passage 1 in assays. Primary juvenile foreskin HDMECs were purchased from Promocell and cell numbers expanded in culture until passage 6 before being used in assays (passage 6–8). Both cell types showed coexpression of both CD31 and CD105 (Figure A in Supplementary Material)

### PBMC Preparation and Cell Sorting of CD4^+^ T Cells and Tregs

Whole blood was collected from healthy donors with signed consent following ethically approved protocols (LREC Ref: 07/H0504/93) into BD Vacutainer^®^ K2E tubes (BD Biosciences, UK). PBMCs were isolated from whole blood using density gradient sedimentation techniques (Lymphoprep™, Axis-Shield, UK) after dilution in sterile PBS. Buffy coats were harvested, washed, and cell counts were conducted. Cells were resuspended in PBS + 1% bovine serum albumin for cell surface staining.

PBMCs were stained with PerCP-Cy5.5 conjugated anti-CD4 (RPA-T4) mAb, PE conjugated anti-CD25 (CD25-3G10) mAb, and eFluor 450 conjugated anti-CD127 (eBioRDR5) mAb for 30 min at 4°C. Cell sorting was conducted using BD FACSAria Cell Sorter (BD Bioscience, UK). CD4^+^ T cells were gated as live singlet CD4^+^ T cells, Tregs were gated as live singlet CD4^+^CD25^hi^CD127^low^ T cells and Teff were gated as live singlet CD4^+^CD25^−^ T cells. Cells were sorted in to RPMI culture media, washed, and used immediately in functional assays. For FOXP3 analysis, cells were fixed and permeabilized using the FOXP3/Transcription factor staining buffer set (eBioscience, UK) and stained with APC conjugated anti-FOXP3 (PCH101) mAb for 30 min at 4°C.

### Coculture of CD4^+^ T Cells and Human ECs

Confluent ECs were plated onto gelatinized 24-well culture plates at 1 × 10^5^ cells/well and cultured overnight to achieve confluence. ECs were used unstimulated or stimulated with 10 U/mL IFN-γ or 1 ng/mL TNF-α for 24 h. Isolated CD4^+^ T cells were stained with 5 µM CFSE. ECs were washed twice with warm RPMI media to remove stimulating cytokine media and CFSE-stained CD4^+^ T cells were plated at 1 × 10^5^ cells/well an EC-CD4 ratio of 1:1. Cocultures were stimulated with 3 µg/mL PHA or 5 µg/mL aCD3 and 10 µg/mL aCD28 incubated together for 72 or 120 h. Appropriate controls of unstimulated EC or CD4^+^ T cell alone cultures and cocultures were also included.

At 72 or 120 h, cells and supernatants were collected and adherent cells were trypsinised and collected into FACS tubes. LIVE/DEAD fixable violet dead cell stain (405 nm; Life Technologies, UK) was added to allow for dead cell exclusion and cells were stained with antibodies for CD4, CD25, and FOXP3 expression. Flow cytometric analysis of the cocultures was completed on the BD FACSAria and data were analyzed using FlowJo software v7.6.5.

### Coculture of ECs and Tregs and Treg Suppression Assay

Confluent ECs were seeded at 1 × 10^5^ cells/well 24 h before use in 24-well plates and left either unstimulated or stimulated separately with 10 U/mL IFN-γ and 1 ng/mL TNF-α or in combination for 24 h prior to coculture with T cells. CD4^+^CD25^hi^CD127^low^ cells (Tregs), CD4^+^CD25^−^ cells (Teffs) and CD4^+^ T cells were isolated from PBMCs using cell sorting as described. ECs were washed to remove exogenous cytokines and Tregs (30,000 cells/well) were added and cocultured with ECs in complete RPMI media for a further 24 h. Transwell assays were also conducted where Tregs were plated on to the upper chamber of a Transwell (Corning 3470 Transwell; pore size 0.4 µm; Corning Life Sciences) suspended above a monolayer of ECs. On the same day, Teffs and CD4^+^ cells were stained with 5 µM CFSE and rested for 24 h whereas accessory cells (47 Gy gamma-irradiated autologous PBMCs) were plated at 1 × 10^5^ cells/well with plate-bound aCD3 antibody (2.5 µg/mL) in 96-well round-bottom plates (kept in 100 µL complete RPMI media) for the same 24 h.

After 24 h coculture, Tregs were recovered by gentle washing of EC monolayers with complete RPMI media and supernatants with detached Tregs were collected. CFSE-stained Teffs and CD4^+^ cells were also collected from culture plastic where they had been allowed to incubate for an equivalent time as Tregs with ECs. CFSE-stained Teffs or CD4^+^ T cells were then plated with the autologous aCD3-stimulated accessory cells at 25,000 cells/well and Tregs were added to Teffs at a ratio of 1:1 in the wells, with appropriate controls. After a further 72 h incubation, cells were harvested and stained with LIVE/DEAD and for CD4 and CD25 expression, then fixed and permeabilized for FOXP3 intracellular staining. Expression of PD-1 and OX40 on Tregs was also interrogated *via* staining cells with PE-Cy7 conjugated PD-1 (EH12.2H7) mAb and Brilliant Violet 421 conjugated OX40 (Ber-ACT35) mAb for 30 min at 4°C. Cells were analyzed by flow cytometry for Teff proliferation as indicated by dilution of CFSE fluorescence and antigen expression on the BD FACSAria. Data was analyzed using FlowJo software v7.6.5.

### Cytokine Quantification

To detect possible soluble mediators, HUVECs or HDMECs were plated on to gelatinized 24-well plates at 1 × 10^5^ cells/well and cultured overnight to reach confluence. Cells were stimulated for 24 h with either 10 U/mL IFN-γ, 1 ng/mL TNF-α or a combination of both cytokines with appropriate controls. Cell monolayers were then washed twice and media was replaced with 1 mL warm complete RPMI media. Cells were cultured for a further 24 h before the conditioned media were collected. Conditioned media were analyzed for cytokine, chemokine, and growth factor using ELISA or multiplex-based assays [Luminex^®^ performance assay human cytokine panel base kit A (LUH000) and individual bead sets, Human granulocyte colony-stimulating factor (G-CSF) DuoSet^®^ ELISA development systems (R&D systems), Human IL-6 ELISA Ready-SET-Go! ^®^, and Human granulocyte macrophage colony-stimulating factor (GM-CSF) ELISA Ready-SET-Go! ^®^ (2^nd^ Generation) (eBioscience, UK)].

### Statistical Analyses

Statistical significance was determined by carrying out statistical analysis using the GraphPad Prism 6 software (GraphPad Software, Inc.; La Jolla, CA, USA). One-way analysis of variance (ANOVA) was used when comparing three or more groups, whereas two-way ANOVA analysis was used for data with two affecting factors. Appropriate *post hoc* tests were completed, including Dunnett’s, Tukey, and Sidak methods, where mentioned.

## Results

### Human ECs Support T Cell Proliferation

**Figure 1:**
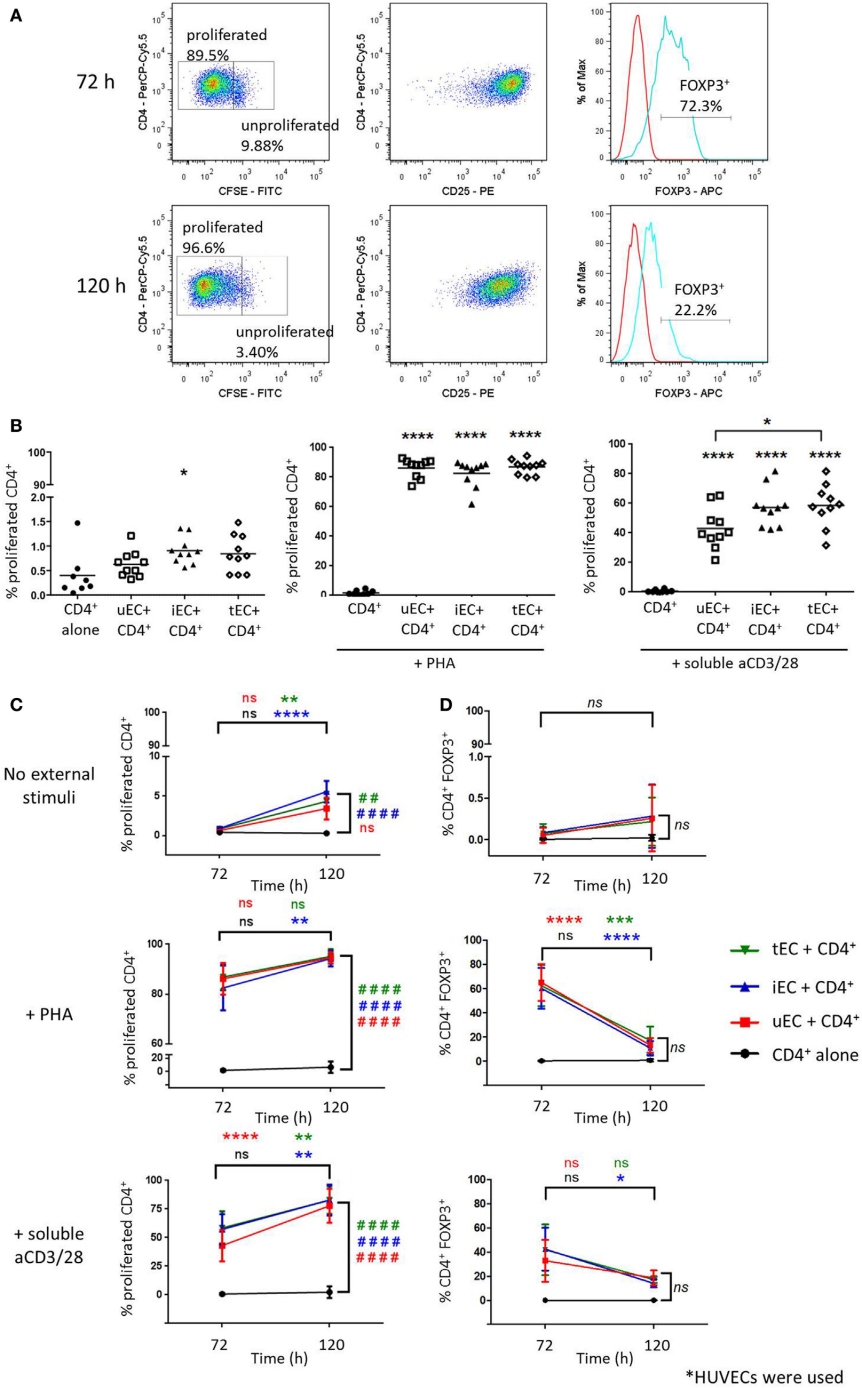
Proliferation of CD4^+^ T cells in endothelial cell (EC) cocultures and expression of FOXP3 of proliferated CD4^+^ T cells. Cells were harvested from cocultures of CD4^+^ T cells with HUVECs at 72 or 120 h and analyzed by flow cytometry, where T cells were discriminated based on CD4 expression. **(A)** CD4^+^ T cells with the highest CFSE fluorescence were gated as unproliferated whereas those with diluted/low CFSE fluorescence were gated as the proliferated population. The CD25 and FOXP3 expression of proliferated cells (blue line) were analyzed with the aid of the isotype control (red line). **(B)** Proliferation of CD4^+^ T cells cultured with unstimulated (uEC) or IFN-γ (iEC) or tumor necrosis factor (TNF)-α-stimulated (tEC) HUVECs were analyzed by CFSE dilution at 72 h, in which coculture had no external stimuli or were stimulated with phytohemagglutinin (PHA) or aCD3/28 antibodies. One-way analysis of variance (ANOVA) with Tukey test was conducted; ****P < 0.0001, *P < 0.05 cf. control; *n* = 10. **(C)** Comparison of CD4^+^ T cell proliferation in cocultures with uEC, iEC, or tEC at 72 and 120 h, in which cocultures had no external stimuli or were stimulated with PHA or aCD3/28 antibodies. Two-way ANOVA analysis with Tukey test was conducted; ****P < 0.0001; **P < 0.01 for 120 h cf. 72 h data; ^####^*P* < 0.0001, ^##^*P* < 0.01 cf. control for 120 h data; *n* = 10/6. **(D)** FOXP3 expression of the proliferated CD4^+^ T cell populations at 72 and 120 h from the different HUVEC-CD4^+^ T cell cocultures were compared, where data are expressed as percentage of CD4^+^FOXP3^+^ cells from the total number of CD4^+^ T cell analyzed, with mean ± SD shown. Two-way ANOVA with Tukey test was conducted, where ****P < 0.0001, ***P < 0.001, *P < 0.05 for data at 120 h cf. data at 72 h; *n* = 4.

Cocultures of CSFE-labeled CD4^+^ T cells with human ECs were analyzed at 72 and 120 h as shown in Figure 1A, where cells with low CFSE fluorescence were gated as proliferated cells and expression of markers including CD25 and FOXP3 were measured. Initial experiments showed that in the absence of any accessory cells, CD4^+^ T cells did not proliferate in response to PHA or soluble aCD3/28 (Figure 1B). When CD4^+^ T cells were cocultured with human ECs for 72 h in the absence of mitogens, low but significant levels of proliferation were observed in cocultures with IFN-γ-stimulated ECs. Similarly, when CD4^+^ T cells were allowed to interact with human ECs for 120 h, significant CD4^+^ T cell proliferation was seen in the absence of mitogens only in cocultures where the EC had been previously stimulated by IFN-γ or TNF-α (Figure 1C). In the presence of PHA or soluble aCD3/28 and EC acting as accessory cells, high levels of CD4^+^ T cell proliferation were observed (*P* < 0.0001, *n* = 10).

These cells were interrogated for their intracellular FOXP3 expression. In control CD4^+^ T cell HUVEC cocultures, with no added mitogen, there was no significant FOXP3 expression in CD4^+^ T cells observed at either 72 or 120 h of culture nor was this seen when cytokine stimulated HUVEC were used as accessory cells (Figure 1D). However, high expression levels of FOXP3 were seen with proliferated T cells in mitogen-stimulated CD4^+^ T cell HUVEC cocultures at 72 h with both unstimulated and cytokine stimulated HUVECs. This FOXP3 expression however, was transient because at 120 h the expression levels in cocultured, mitogen activated proliferated CD4^+^ T cells was significantly reduced compared to 72 h and was similar to the levels seen in control mitogen-free cultures.

**Figure 2:**
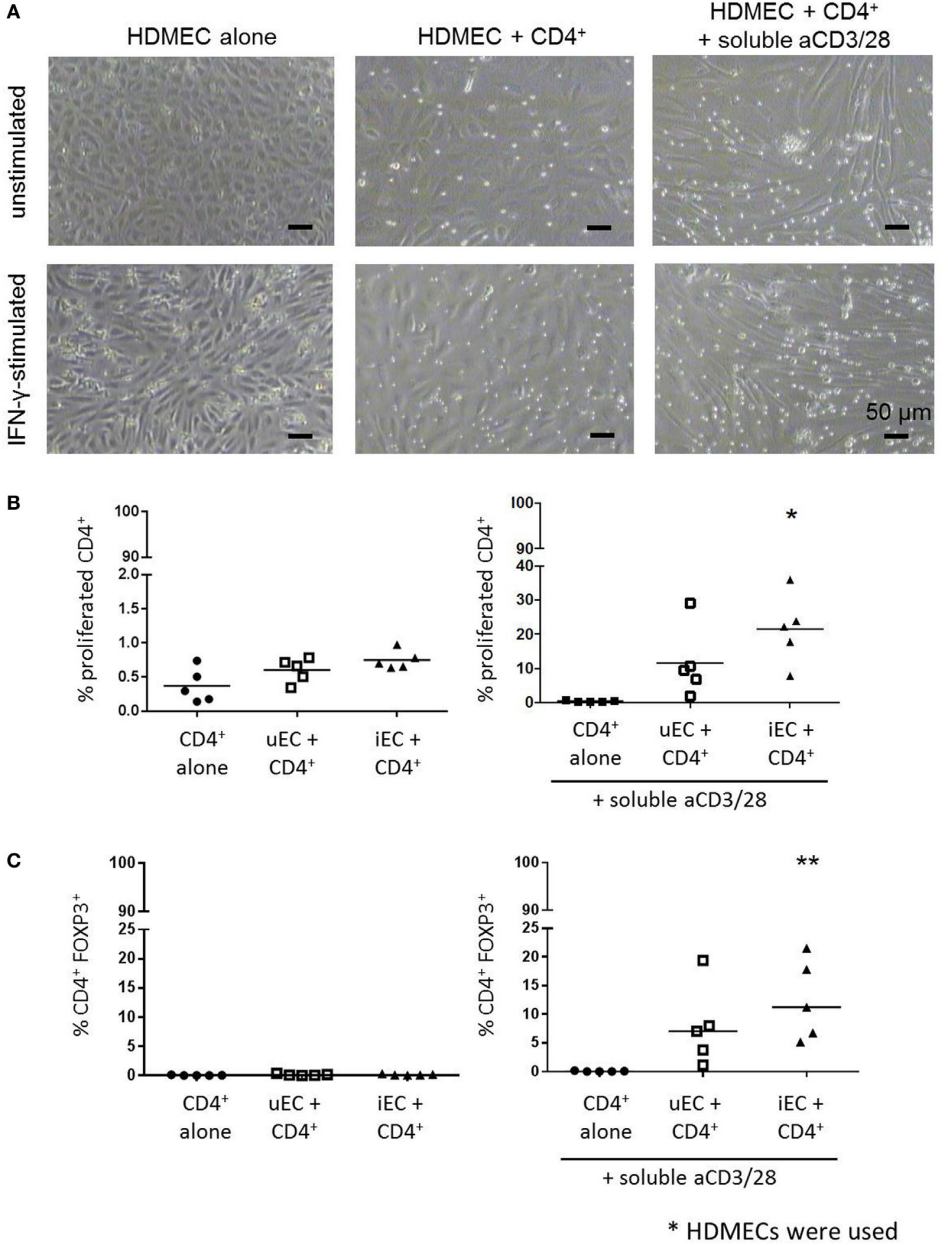
Proliferation of CD4^+^ T cells in HDMEC cocultures and their expression of FOXP3. **(A)** Phase contrast micrographs of HDMEC-CD4^+^ T cell cocultures using unstimulated or interferon (IFN)-γ-stimulated HDMECs in the absence or presence of stimulatory aCD3/28 antibodies were taken at 72 h; micrographs were taken at 100× original magnification; scale bar of 50 µm included. **(B)** CFSE-labeled CD4^+^ T cells were cocultured with HDMECs used either unstimulated (uEC) or stimulated with 10 U/mL IFN-γ (iEC) for 24 h; proliferation of CD4^+^ T cells was analyzed by CFSE dilution at 72 h. Proliferation of CD4^+^ T cells in HDMEC-CD4^+^ T cells cocultures stimulated with aCD3/28 antibodies were also measured. One-way analysis of variance (ANOVA) with Dunnett’s test was conducted; *P < 0.05 cf. control, *n* = 5. **(C)** FOXP3 expression of proliferated CD4^+^ T cells from the same HDMEC-CD4^+^ T cell cocultures was also analyzed at 72 h, where data are expressed as percentage of CD4^+^FOXP3^+^ cells from the total CD4^+^ population with means shown. One-way ANOVA with Dunnett’s test was used; **P < 0.01 cf. control; *n* = 5.

Cocultures of CFSE-stained CD4^+^ T cells with HDMECs with or without aCD3/28 were conducted (Figure 2A). In the absence of external stimulation, there was no significant T cell proliferation in these cocultures by 72 h (Figure 2B). In cocultures of CD4^+^ T cells and IFN-γ-stimulated HDMECs with soluble aCD3/28, there was a significant proliferation of CD4^+^ T cells. A significant increase in FOXP3 expression was also found when CD4^+^ T cells were cocultured with IFN-γ-stimulated HDMECs and soluble aCD3/28 but not in control non-cytokine stimulated cocultures (Figure 2C). Additionally, IFN-γ-activated HUVECs were capable of supporting proliferation of purified Teff and Treg populations separately in the presence of soluble aCD3/28 (Figure B in Supplementary Material).

### Activation of Treg Suppressor Function by HUVEC Is by Both Contact-Dependent and Independent Mechanisms

**Figure 3:**
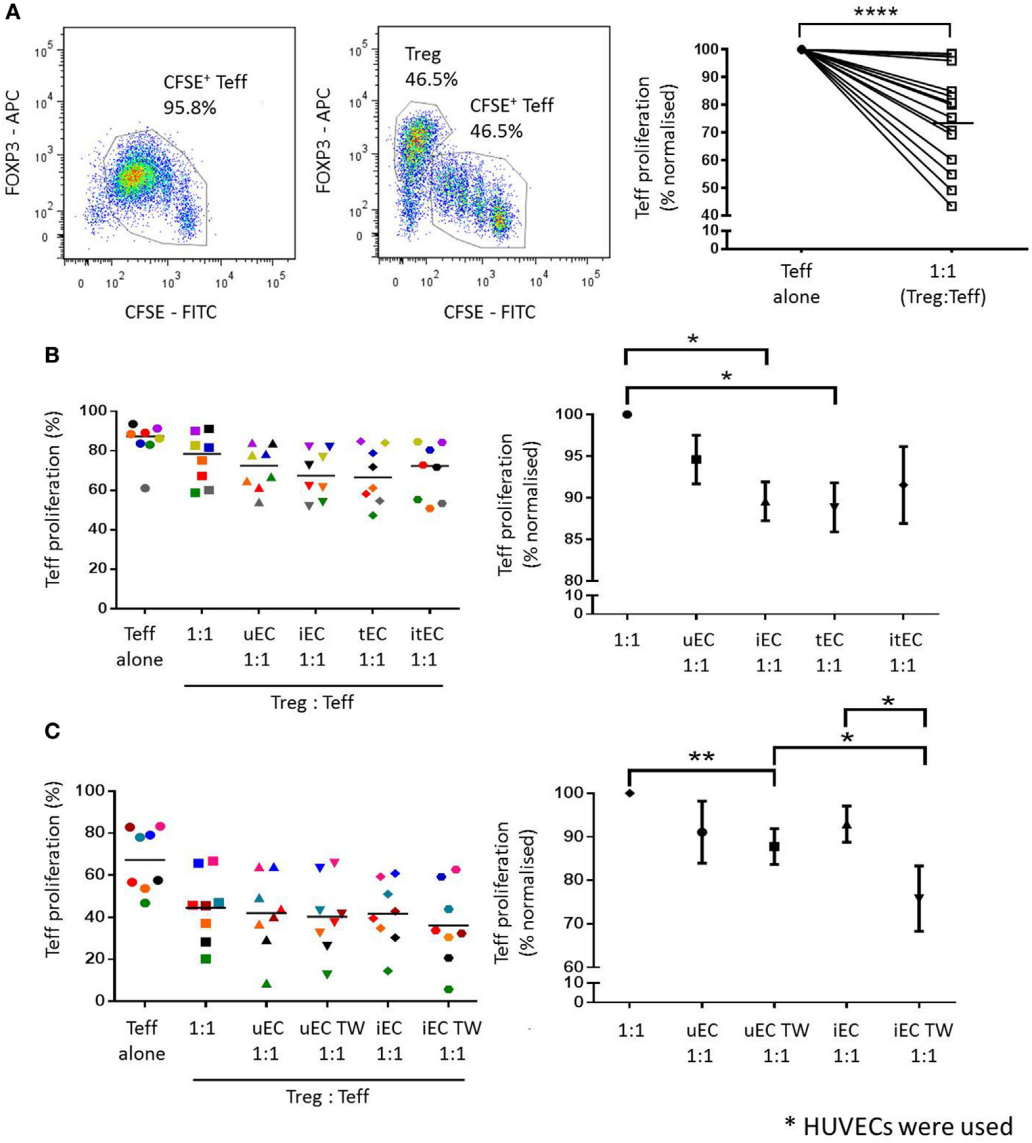
Enhancement of Treg suppressor function after coculture with activated human umbilical vein ECs (HUVECs). **(A)** Treg suppression assays were set up where CFSE-labeled Teffs were either stimulated with 5 µg/mL plate-bound aCD3 antibody (in the presence of gamma-irradiated autologous PBMCs) or with CD4^+^CD25^hi^CD127^low^ Tregs at a 1:1 ratio. Teff proliferation was analyzed by CFSE dilution and the suppressive capacity of Tregs was measured by comparing the reduction of Teff proliferation in the presence of Tregs. Tregs from 16 different donors were found to suppress Teff proliferation in varying capacity. Two-tailed paired T test was conducted; ****P < 0.0001; *n* = 16. **(B)** CD4^+^CD25^hi^CD127^low^ cells (Tregs) were cocultured with unstimulated (uEC), interferon (IFN)-γ- (iEC), tumor necrosis factor (TNF)-α- (tEC), or both IFN-γ and TNF-α (itEC) HUVECs for 24 h, before they were recovered and plated in a Treg suppression assay to measure suppressive capacity. Raw data is expressed as % proliferated Teff, with each experiment represented by a different color. Data are also expressed where Teff proliferation is normalized to proliferation in 1:1 cultures as 100%, showing mean ± SEM. One-way analysis of variance (ANOVA) with Dunnett’s test was used; *P < 0.05, *n* = 8. **(C)** The contact-dependent nature of the modulation of Treg function by HUVEC was investigated where Tregs were plated in direct contact with uEC or iEC, or suspended above unstimulated (uEC TW) or IFN-γ-stimulated (iEC TW) HUVECs in Transwells for 24 h, before plating in a Treg suppression assay. Raw data are expressed as % proliferated Teffs, with each experiment represented by a different color. Data are also expressed where Teff proliferation is normalized to proliferation in 1:1 cultures as 100%, showing mean ± SEM. One-way ANOVA with Dunnett’s test was used; **P < 0.01, *P < 0.05, *n* = 8.

Cocultures of HUVECs with Tregs were conducted, where CD4^+^CD25^hi^CD127^low^ cells were plated in direct cocultures with unstimulated (uEC, 1:1 ratio), IFN-γ-stimulated (iEC, 1:1 ratio), TNF-α-stimulated (tEC, 1:1 ratio), or both IFN-γ + TNF-α-stimulated (itEC, 1:1 ratio) HUVECs, for 24 h, then Tregs recovered and plated in a suppression assay. Purity of each population is shown in supplementary Figure C in Supplementary Material. In an assay of Treg function, despite some variation in the suppression by Tregs from different donors, control Tregs which had not been cultured with ECs suppressed Teff proliferation in the expected manner (Figure 3A, *P* < 0.0001; *n* = 16).

After direct coculture with HUVECs, Tregs were shown to have an enhanced suppressor function as indicated by a decrease in Teff proliferation compared to controls where Tregs were not previously cocultured with HUVEC (Figure 3B). Tregs cocultured with unstimulated HUVECs (uEC 1:1) produced an additional mean reduction of 4.4% (normalized to individual control 5.4%) in Teff proliferation compared to control Tregs (1:1) in the absence of ECs. Tregs cocultured with IFN-γ- (iEC 1:1) or TNF-α-stimulated (tEC 1:1) HUVECs showed a further enhancement in their suppressive capacity causing an 8.0% (normalized 10.4%) and 8.2% (normalized 11.2%) further reduction in Teff proliferation compared to control Tregs (1:1) (*P* < 0.05; *n* = 8). Tregs cocultured with IFN-γ plus TNF-α-stimulated HUVECs also showed increased suppressive function compared to unstimulated Treg but was not statistically significant compared to the suppressor function of Tregs cocultured with HUVEC stimulated with IFN-γ or TNF-α alone.

The requirement for direct contact on the modulation of Treg function by HUVEC was next investigated using purified and isolated Tregs cultured in Transwells suspended above control or cytokine-activated ECs. Following coculture of Tregs either in contact with or suspended above control ECs, the suspended Tregs showed a small but non-significant (1.8%, normalized 3.3%) reduction in Teff proliferation (Figure 3C). However, in Tregs previously suspended over IFN-γ stimulated HUVEC, a significantly greater reduction in Teff proliferation of 5.6% (normalized 17.1%) was demonstrated compared to direct contact coculture of Tregs with IFN-γ-stimulated EC (*P* < 0.05; *n* = 8). In the contact-independent conditions, Tregs previously suspended above IFN-γ stimulated HUVECs showed a greater suppressive effect on Teff proliferation than those suspended above unstimulated HUVECs [increase of a further 4.2% (normalized 12.0%) (*P* < 0.05; *n* = 8)].

### Activation of Treg Suppressor Function by HDMEC Is *via* a Contact-Independent Mechanism

After direct coculture of Tregs with unstimulated HDMECs (uEC 1:1; Figure 4A), the suppressive capacity of Tregs on Teff proliferation was not significantly increased when compared to control Tregs (1:1). This was also the case for Tregs cultured on Transwells above unstimulated HDMECs (uEC TW 1:1). When Tregs were cocultured directly with IFN-γ-stimulated HDMECs, their suppressive capacity was not enhanced compared to control Treg suppressor function. However, Tregs cultured in Transwells above previously IFN-γ-stimulated HDMECs showed an increase in their suppressor function compared to those Tregs in direct contact with previously IFN-γ-stimulated HDMEC. Teff proliferation was suppressed by these suspended Tregs by 8.2% (17.9% normalized to control 1:1) (*P* < 0.05; *n* = 8).

**Figure 4:**
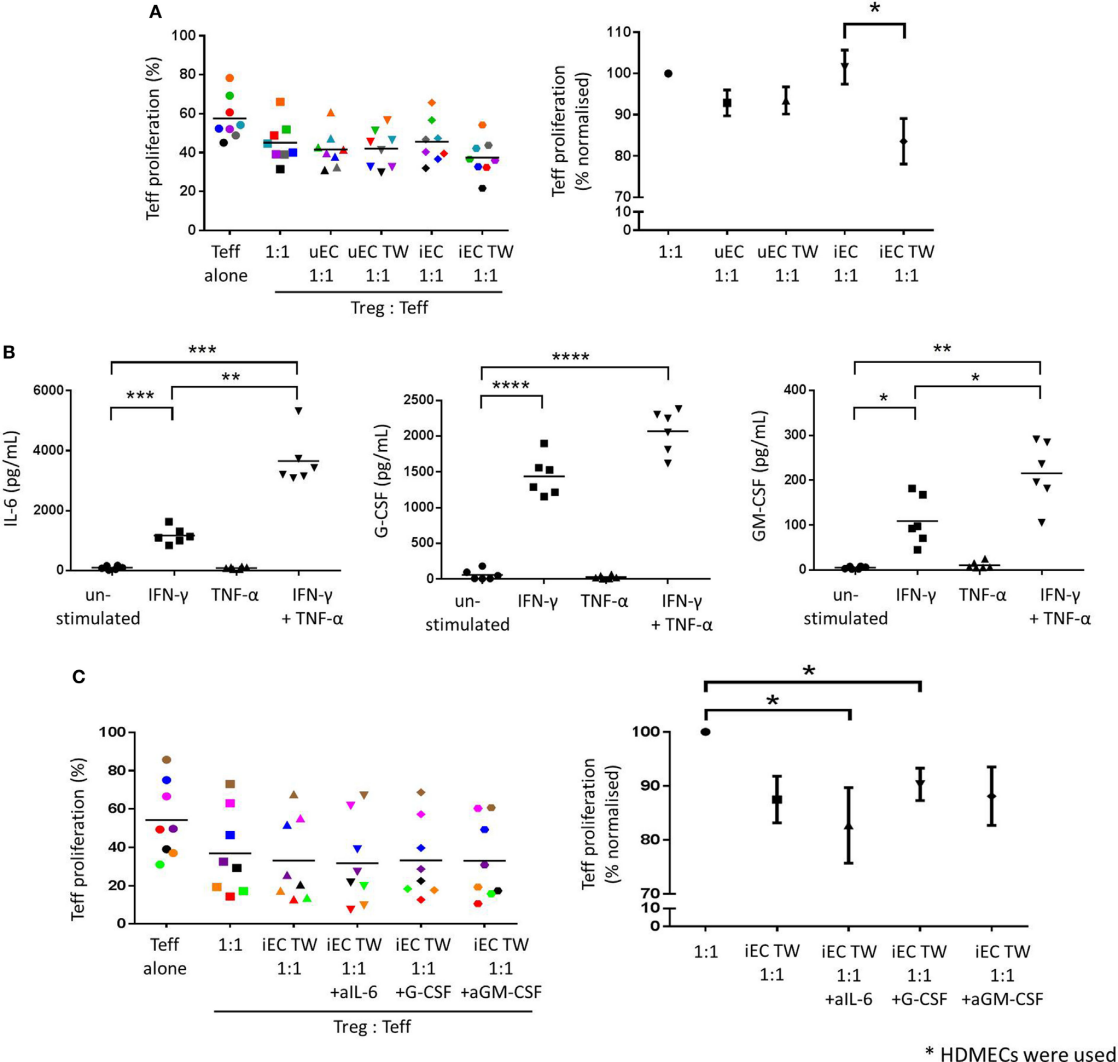
Increased Treg suppressive capacity through contact-independent interaction with activated HDMECs. **(A)** The effect of HDMEC interaction on Treg function was investigated whereby CD4^+^CD25^hi^CD127^low^ were plated in direct contact with unstimulated (uEC) or IFN-γ-stimulated (iEC) HDMECs, or suspended above unstimulated (uEC TW) or IFN-γ-stimulated (iEC TW) HDMECs in Transwells for 24 h, then recovered and plated in Treg suppression assays. Data are expressed as % proliferated Teffs, with each experiment represented by a different color. Data are also expressed where Teff proliferation is normalized to proliferation in 1:1 cultures as 100%, showing mean ± SEM. One-way analysis of variance (ANOVA) with Dunnett’s test was used; *P < 0.05; *n* = 8. **(B)** HDMECs were left unstimulated or stimulated with 10 U/mL IFN-γ, 1 ng/mL TNF-α, or a combination of both cytokines for 24 h. Cells were washed and media replaced with 1 mL complete RPMI. Conditioned media was collected after 24 h and secreted cytokines detected using bead-based multiplex assays or ELISAs. Production of IL-6, G-CSF, and GM-CSF by HDMECs are shown with means drawn. One-way ANOVA with Dunnett’s test was conducted; ****P < 0.0001, ***P < 0.001, **P < 0.01, and *P < 0.05 cf. control; *n* = 6. **(C)** Tregs were cocultured in the upper chambers of Transwells with IFN-γ-stimulated (iEC TW) HDMECs in the lower chambers for 24 h, where 5 µg/mL of anti-IL-6, anti-G-CSF, or anti-GM-CSF blocking antibody was added in the lower chambers. Tregs were recovered and plated in a Treg suppression assay where Teff proliferation was measured by CFSE dilution. Data are expressed as % proliferated Teffs, with each experiment represented by a different color. Data are also expressed where Teff proliferation is normalized to proliferation in 1:1 cultures as 100%, showing mean ± SEM. One-way ANOVA with Dunnett’s test was used; *P < 0.05; *n* = 8.

### Stimulated ECs Produce IL-6, GM-CSF, and G-CSF, but These Are Not Responsible for Enhanced Treg Suppressor Function

To study potential factors responsible for the indirect nature of the enhanced Treg suppressor function, conditioned media from HDMECs was analyzed using cytokine multiplex assay and ELISAs. Of all the cytokines produced by ECs, (Figure D in Supplementary Material) only IL-6, G-CSF, and GM-CSF were modulated by IFN-γ; therefore, these factors were studied in greater detail (Figure 4B). Unstimulated HDMEC cultures produced low levels of IL-6, G-CSF, and GM-CSF, whereas IFN-γ stimulation significantly increased IL-6 (1,166 ± 274.1 pg/mL, *P* < 0.001; *n* = 6), G-CSF (1,439 ± 278.1 pg/mL, *P* < 0.0001; *n* = 6), and GM-CSF (108.9 ± 54.2 pg/mL, *P* < 0.05; *n* = 6). Production of these cytokines was not significantly stimulated by TNF-α alone; however, stimulation with both IFN-γ and TNF-α in combination significantly increased levels of IL-6, G-CSF, and GM-CSF, compared to unstimulated controls. Furthermore, IL-6 and GM-CSF secretion was significantly elevated above the levels stimulated by IFN-γ alone. Combined cytokine treatment did not, however, significantly increase G-CSF secretion above that of IFN-γ alone.

The function of the soluble mediators IL-6, G-CSF, and GM-CSF on the interaction between HDMEC and Tregs, in terms of the modulation of Treg suppressive capacity, was investigated by the use of blocking antibodies specific for each of the three cytokines. Blocking antibodies against IL-6, G-CSF, or GM-CSF were added at 5 µg/mL in the lower chamber with the IFN-γ-stimulated HDMEC in the Treg-EC cocultures followed by assays of Treg suppressor function with appropriate isotype controls. Inclusion of blocking antibodies against IL-6 appeared to significantly increase Treg suppressor function as shown with the greater decrease in Teff proliferation in Figure 4C, when compared to control Tregs (1:1) (*P* < 0.05; *n* = 8). However, this was not significant when compared with Tregs cocultured above IFN-γ-stimulated HDMECs alone. Blockade of G-CSF or GM-CSF in indirect coculture of Tregs with IFN-γ-stimulated HDMECs had no significant effect on Treg suppressive capacity against Teffs proliferation.

### IFN-γ but Not TNF-α Enhances EC PD-1 Ligands PD-L1 and PD-L2

**Figure 5:**
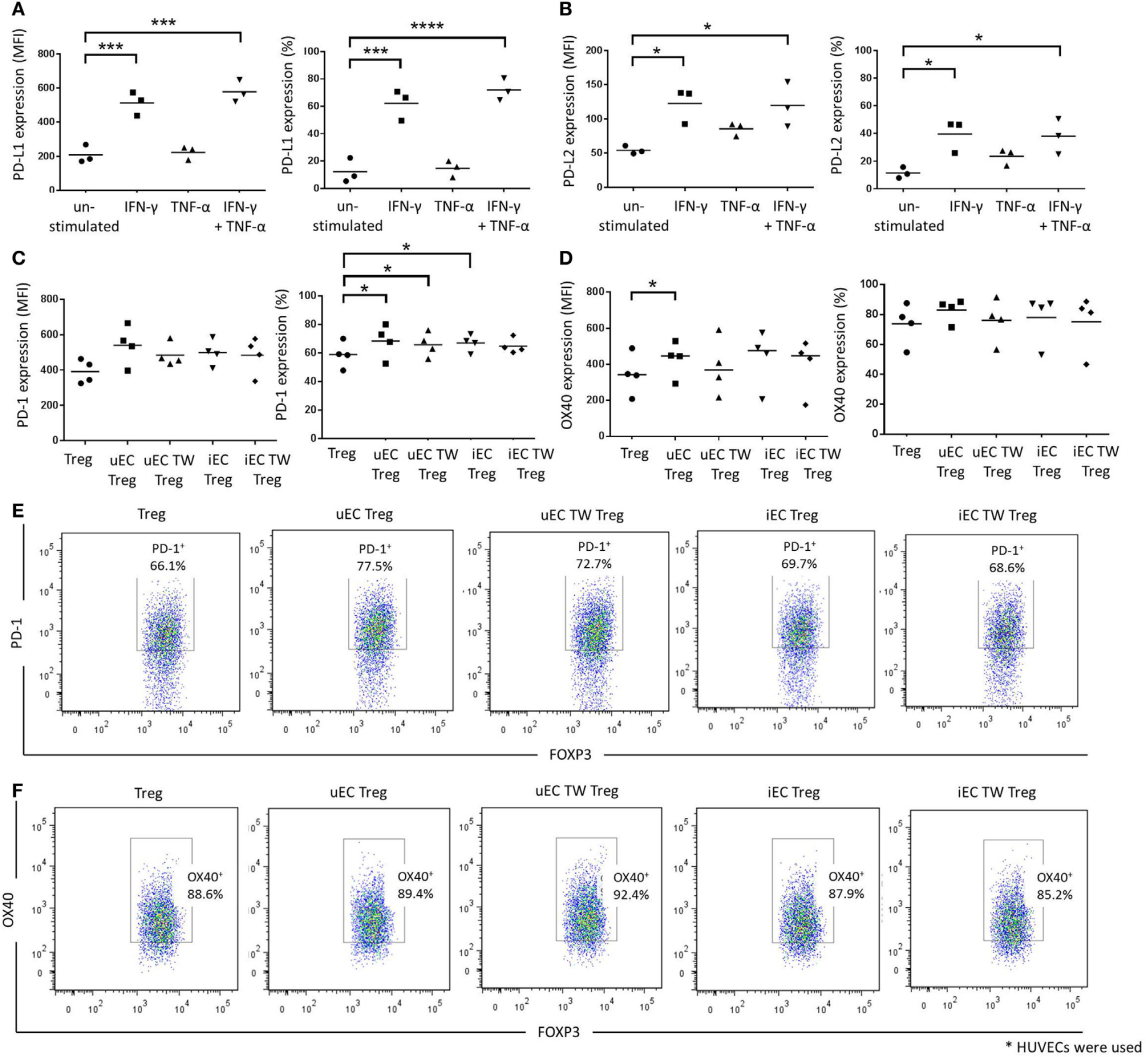
Involvement of PD-1/programmed death ligand (PD-L)1/2 in the contact-dependent interactions between HUVEC and Tregs. Human umbilical vein ECs (HUVECs) were stimulated with 10 U/mL IFN-γ, 1 ng/mL tumor necrosis factor (TNF)-α, or combination of both cytokines for 24 h. Cells were then stained and analyzed for **(A)** PD-L1 or **(B)** PD-L2 expression using flow cytometry. Data are expressed as mean fluorescence index (MFI) and percentage of positive cells, with means shown. One-way analysis of variance (ANOVA) with Dunnett’s test was conducted; ****P < 0.0001, ***P < 0.001, *P < 0.05; *n* = 3. Modulation of Treg phenotype after HUVEC interactions were also investigated whereby Tregs were plated in direct contact with unstimulated (uEC Treg) or IFN-γ-stimulated (iEC Treg) HUVECs, or in the upper chambers of Transwells with unstimulated (uEC TW Treg) or IFN-γ-stimulated (iEC TW Treg) HUVECs. Tregs were then recovered and plated in a Treg suppression assay and analyzed for **(C)** PD-1 and **(D)** OX40 expression after 72 h using flow cytometry. One-way ANOVA with Dunnett’s test was conducted; *P < 0.05; *n* = 3. Plots of a representative experiment are shown in Figures **(E,F)**.

PD-1 and its ligands have been implicated in the interaction between Tregs and ECs or other cell types, which can lead to activation of Treg function or induction of Treg populations (14, 16). In our system, unstimulated HUVECs showed low levels of PD-L1 and PD-L2 expression, whereas IFN-γ stimulation caused significant increases in expression of both ligands on HUVECs, indicated by both their mean fluorescence index (MFI) values and percentages of positive cells (*P* < 0.001 or *P* < 0.05 cf. unstimulated control; *n* = 3) (Figures 5A,B). In contrast, TNF-α treatment of HUVECs did not affect either PD-L1 or PD-L2 expression. However, PD-L1 and PD-L2 expression was increased significantly on HUVECs with combined IFN-γ and TNF-α cytokine stimulation but not significantly above IFN-γ alone cultures suggesting only IFN-γ regulated PD-L1 and -2 expression.

### EC–Treg Interaction Changes Treg Expression of PD-1 and OX40

The expression of costimulatory molecules PD-1 and OX40 on Tregs after HUVEC coculture was examined as part of the possible mechanism of action in the Treg suppression assay. There were no significant changes in PD-1 MFI values for Tregs regardless of coculture conditions with HUVECs (Figures 5C,E). However, the percentage of PD-1^+^ Tregs was increased in Tregs that were in direct contact with unstimulated HUVECs (uEC Treg) or IFN-γ-stimulated HUVECs (iEC Treg) or in Transwells above unstimulated HUVEC (uEC TW Treg) (*P* < 0.05; *n* = 5). This was not observed, however, in Tregs that were cocultured above IFN-γ-stimulated HUVECs (iEC TW Treg). In contrast, there was a slight increase in OX40 MFI values in Tregs that were in direct contact with unstimulated HUVECs (uEC Treg) (*P* < 0.05; *n* = 4) but there was no difference in OX40 expression profiles of Tregs (MFI values and percentage of positive cells) for other HUVEC coculture conditions (Figures 5D,F).

## Discussion

Endothelial cells can promote CD4^+^ T cell activation and proliferation in the absence of professional antigen presenting cells. In the presence of an external mitogen including PHA or stimulatory aCD3/28 antibodies, strong proliferation was shown with CD4^+^ T cells in cocultures with the endothelium regardless of prior EC activation with inflammatory cytokines. This immunological function, seen with ECs from different lineages suggests a further functional role for the endothelium in immune cell activation. In the absence of any external mitogen, T cell proliferation occurred only in cocultures of CD4^+^ T cells with IFN-γ activated ECs which supports previous literature that demonstrates the ability of ECs in the activation, cytokine production, and proliferation of CD4^+^ T cells (19, 20). Although ECs increased FOXP3 expression within the previously CD25 −ve FOXP3 −ve population, the temporal nature of the FOXP3 expressing population suggests that these cells are likely to represent activated effector T cells which can express FOXP3 without acquiring a regulatory phenotype (21). Taken in conjunction with previous work on ECs, this suggests that the endothelium can provide partial activating signals that may be further augmented by direct contact or cytokine activation from stromal cells (including immune cells) following transmigration into the tissue.

Previous research has shown that rapamycin activated EC can enhance EC dependent Treg suppressor function (16) and that TGF-β and retinoic acid along with rapamycin can induce human iTregs with superior suppressive activity (22). Our data using purified Tregs further demonstrate a role for human ECs in enhancing Treg suppressor function. Both direct cocultures and indirect cocultures of Tregs with cytokine-activated HUVECs enhanced the suppressive capacity of Tregs on Teff proliferation. This augmented Treg activity was greatest in the indirect coculture of IFN-γ-stimulated HUVECs and Tregs and, in the case of HDMECs, the higher Treg suppressive function was only observed in the indirect cultures. This suggests a potentially complex interplay between ECs and Tregs when both cells are in direct contact and that some of the direct interactions may regulate or indeed negate the stimulatory effects of ECs on Tregs. The differences in Treg activation between HUVECs and HDMECs may reflect the nature of the endothelial response to cytokine and the vascular bed from which the cells are derived. Protecting the fetus from insult including the mothers own immunity may explain why HUVEC possess both contact-dependent and independent mechanisms to regulate Tregs and may have evolved to regulate maternal–fetal tolerance (23). In contrast, Tregs in the skin often reside in the tissue (24) and have a greater diversity of roles in relation to resolution of inflammation after exogenous and endogenous stimuli. Interestingly, a previous study also showed that HMEC-1 cells and HDMECs were able to expand Treg populations (15), providing additional support for a role of the skin endothelium in Treg activity. Overall, the combination of murine and human EC studies, including our data, suggests an adaptive immune function for ECs above the simple recruitment of T cells from the circulation.

Roles for the involvement of ECs (HUVECs) in upregulating GITR, CTLA-4, and PD-1 on Tregs (25), antigen presentation, and co-stimulation of T cells by the endothelium (1–4), the suppression of endothelial function by Tregs (26) and Treg inhibition of Teff recruitment that defines the kinetics of an immune response (27) have been reported. PD-1 (and its ligands PD-L1 and PD-L2) and OX40 have also been studied as targets for cancer immunotherapy to boost antitumor immunity (28). The PD-1 signaling pathway has also been implicated in EC–Treg interactions (14, 16) and increased PD-1 expression on Tregs after EC modulation was observed in the current study which concurs with the work of Chen et al. (25). The exact role for PD-1 on Tregs has been debated, with evidence to support both PD-1 promoted Treg function and inhibition of Treg function in other scenarios (8, 25). Our data showing that ECs increased the percentage of PD-1^+^ Tregs and also augmented Treg suppressive activity suggests that EC-induced PD-1 expression on Tregs is likely involved in the increased suppressor function of the Tregs. Furthermore, the upregulation of PD-1 ligands expressed on activated HUVECs suggests that ECs possess the relevant mechanisms to activate Tregs *via* the PD-1/PD-L1 signaling pathway during direct interactions and supports the findings of Wang et al. (16) who were able to show increased Treg differentiation in a rapamycin activated endothelial PD-L1-dependent manner.

A limitation to this present study is that the PD-1/PD-L1 interaction was not studied further with blocking antibodies to better define the role of these ligands in the Treg/EC interaction. These studies will need to be performed to show the definitive role of PD-1/PD-L1 in Treg/EC activity. However, in the light of other research, the nature of the endothelial regulation of PD-1 expression on Tregs suggests that the response may depend partly on the Treg subset in question as well as on other mediators. For instance, in a chronic HCV infection model (25) the PD-1/PD-L1 interaction was shown to lower the expansion of Treg phenotype compared to Teff whereas the same ligand interaction was shown to regulate induced Tregs and helped to maintain their Treg phenotype and suggests a role in Treg plasticity (8). Indeed, Lion et al. (29) have recently shown that ECs can amplify Tregs in a model of alloactivation *via* HLA-DR, the expression of which can be modified by immunosuppressants. Despite this group showing expression of endothelial PD-L1 in their model, immunosuppressants were not shown to affect PD-L1 levels. In addition, a previous study by the same group did not observe a role for PD-L1 in Treg expansion under inflammatory conditions (15). This suggests that complex interactions and potentially multiple pathways exist that are dependent on the microenvironment, vascular bed, and T cell subsets available and requires further study. It is not entirely clear, therefore, that the contact-dependent mechanism is solely due to the PD-1/PD-L1 axis and it is unlikely to describe the full nature of endothelial specific Treg activation especially in the context of the non-contact mediated Treg activation.

*In vivo*, the length of time that T cells interact with the endothelium would be an important factor in considering the mechanisms as detailed here. In *in vitro* flow based assays, T cell residence on the luminal side of the endothelium can last for some considerable time before full transmigration occurs. In the presence of antigen pulsed endothelium, this interaction is significantly slowed in memory/effector T cells (30) suggesting a range of time dependent signaling events prior to transmigration. Following attachment, complete transmigration took up to 2 h and T cells were shown to use invadosome-like processes in their interaction with the endothelium. Migrating T cells may then either remain in the subendothelial environment in close contact with the endothelium or travel further in to tissue and interact with additional stromal cells. Indeed, subendothelial pericytes and smooth muscle have been suggested to present antigen and provide signals that may regulate T cell function and localize them in close proximity to the endothelium (31). Using, confocal intravital microscopy, Deane et al. showed that in a contact sensitivity model, using mice, Tregs were found to accumulate in the inflamed skin by 24 h, peaking at 48 h (32). This suggests a prolonged response where, due to the high vascularity of the skin, the proximity of ECs and Treg is such that the endothelium is likely to influence Treg function over a prolonged period similar to that used in the present study.

On interrogating the contact-dependent and contact-independent nature of Treg activation the initial screening of HUVEC and HDMEC-conditioned media showed IL-6, G-CSF and GM-CSF as potential soluble mediator candidates. These mediators were produced only after IFN-γ stimulation of ECs, indicating their potential specific involvement in the indirect interactions of IFN-γ-stimulated ECs and Tregs. However, individual blockade of these mediators did not abolish the boosted Treg function in indirect coculture with activated HDMECs and suggests that the interaction is multifaceted or *via* further soluble mediators. A role for IL-6 was suggested, however, in contact-independent effects by ECs on Tregs as the IL-6 blocking antibody increased Treg suppressor function during EC–Treg interactions. This is in line with studies which reported that IL-6 can dampen Treg suppressive activity and skew Treg induction toward Th17 differentiation (33, 34). This suggests that IL-6, in combination with other co-stimulator mechanisms, is partly suppressive on Treg function as this effect is lost when both contact signals and IL-6 signaling were removed, leading to the enhancement of Treg function.

Our initial studies (Figure F in Supplementary Material) have ruled out nitric oxide signaling from the endothelium as a soluble mediator despite NO being generated by EC and a known regulator of T cell function (35). Additionally, TGF-β can regulate Tregs and can be secreted by the endothelium either onto the endothelial surface or out into the media (36, 37). In our hands (Figure F in Supplementary Material) the endothelium constitutively secretes TGF-β but this was not enhanced by either of the stimulating cytokines used thus suggesting that TGF-β is unlikely to mediate the EC/Treg suppressive activity at a distance in this model. However, there may be a role for endothelial surface bound TGF-β when EC and Treg are in close proximity and this could link both contact and non-contact-dependent activities. A further possibility for signaling at a distance lies in the release of endothelial microvesicles. These lipid and protein rich vesicles can be induced by inflammatory cytokines (38) and can carry molecules across distances and appear to mediate responses *via* a soluble mediator(s) when in fact, a contact-like effect is in use (39, 40). The discovery of vascular derived microvesicles in disease and their ability to control T cell activity *via* a range of mechanisms including carriage of microRNAs, particularly miRs 146a and 155 and adhesion molecules suggests this hybrid contact/non-contact mechanism as a further possibility (41–45). Indeed, PD-L1 within microvesicles has been observed released from mesenchymal stem cells (46) and microvesicles have been shown to transfer materials between T cells and APCs (47) suggesting a common mechanism of control form a range of cell types.

Human ECs exhibit important functions over and above leukocyte recruitment that contributes to immune homeostasis, with the distinct mechanisms responsible for these immune functions dependent on the vascular bed from which the endothelium is derived. Based on the current study, it is likely that human ECs activated during inflammation modulate both Teff and Treg populations by enhancing their proliferation and suppressive capacity, respectively. It is also possible that dysregulation of EC–Treg interaction could potentially be a source of unregulated inflammation as not only the control of Treg function by EC interaction is important but the reverse situation where EC function and/or phenotype can be controlled by Tregs. Tregs can exert protective effects during inflammation by downregulating production of proinflammatory mediators by ECs, as suggested by He et al., who showed downregulation of LPS-induced VCAM-1, monocyte chemoattractant protein-1 and IL-6 on HUVECs by Tregs (48). Furthermore, Tregs can also affect EC activation and leukocyte recruitment by dampening EC E- and P-selectin expression that subsequently decreased effector T cell adhesion (26, 49). Finally, blockade of E-selectin in a contact sensitivity model in mice showed increased swelling and neutrophil infiltration due to a lack of Treg recruitment (50). Disruption of EC–Treg interaction can, therefore, have wide ranging consequences.

These studies and ours suggest a complex, tripartite interaction of Teff, Treg, and EC with various activating and inhibitory signals passing between them. The competing signals are likely to evolve with the microenvironment to balance tolerance and inflammation. Where perturbation of any of these mediators is sought, an understanding of the consequences of immune modulation strategies is required. While the current study has provided the groundwork for understanding the nature of the interactions (i.e., direct and indirect) between human ECs and Tregs, future studies identifying the exact mechanisms of indirect EC–Treg interaction may have the potential to identify novel targets for modulation of T cell function controlled by the endothelium.

## Nomenclature

**Table:** 

aCD28	Anti-CD28
aCD3	Anti-CD3
aCD3/28	Anti-CD3 and anti-CD28
ANOVA	Analysis of variance
APC	Allophycocyanin
CFSE	Carboxyfluorescein succinimidyl ester
Cy	Cyanine
EC	Endothelial cell
FITC	Fluorescein isothiocyanate
FOXP3	Foxhead Box P3
G-CSF	Granulocyte colony-stimulating factor
GM-CSF	Granulocyte-macrophage colony-stimulating factor
HDMEC	Human dermal microvascular endothelial cell
HMEC-1	Human dermal microvascular endothelial cell-1
HUVEC	Human umbilical vein endothelial cell
ICOS	Inducible T cell costimulator
iEC	IFN-γ-stimulated endothelial cell
IFN	Interferon
MFI	Mean fluorescence intensity
PBMC	Peripheral blood mononuclear cell
PBS	Phosphate buffered saline
PD-1	Programmed death-1
PD-L1/L2	Programmed death ligand-1/2
PE	R-phycoerythrin
PerCP	Peridinin chlorphyll protein
PHA	Phytohemagglutinin
tEC	TNF-stimulated endothelial cell
Teff	Effector T
Th	T helper
TNF	Tumor necrosis factor
Treg	Regulatory T cell
uEC	Unstimulated endothelial cell

## Ethics Statement

This study was carried out in accordance with the recommendations of Southampton & South West Hampshire Research Ethics Committee B with written informed consent from all subjects. All subjects gave written informed consent in accordance with the Declaration of Helsinki. The protocol was approved by the Southampton & South West Hampshire Research Ethics Committee B. Umbilical cords were collected from the Princess Anne Hospital, Southampton, UK from non-complicated natural vaginal births following agreed ethical collection protocols (Local Research Ethical Committee (LREC); Ref: 07/H0502/83). Whole blood was collected from healthy donors with signed consent following ethically approved protocols (LREC Ref: 07/H0504/93).

## Author Contributions

WL, EH, and TM conceived and designed experiments. WL performed the experiments and carried out the acquisition and analysis. MO provided isolated primary cells. WL, EH, and TM interpreted data and wrote the manuscript.

## Conflict of Interest Statement

The authors declare that the research was conducted in the absence of any commercial or financial relationships that could be construed as a potential conflict of interest.
