# Supplementary material for: Human Endothelial Cells Modulate CD4+ T Cell Populations and Enhance Regulatory T Cell Suppressive Capacity
Source: Front Immunol. 2018 Mar 23;9:565. doi: 10.3389/fimmu.2018.00565 (PMC5876242; doi:10.3389/fimmu.2018.00565)
Supplement: Supplementary file 1 [file Data_Sheet_1.DOCX]

**Supplementary Material**

**Figure A: HUVEC and HDMEC phenotype**

**
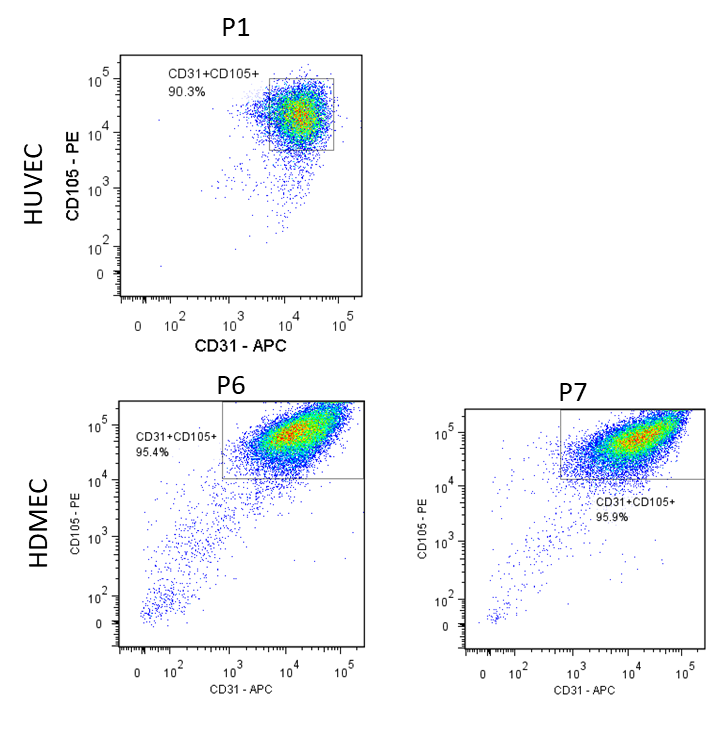
**

Primary ECs isolated from umbilical veins by enzymatic digestion were used in our assays. Using flow cytometry, these cells were shown to express both CD31 and CD105 at passage 1. HDMECs were used at passages 6 and 7, where they also showed uniform co-expression of CD31 and CD105.

**Figure B: Cocultures of HUVECs with Teffs or Tregs**

**
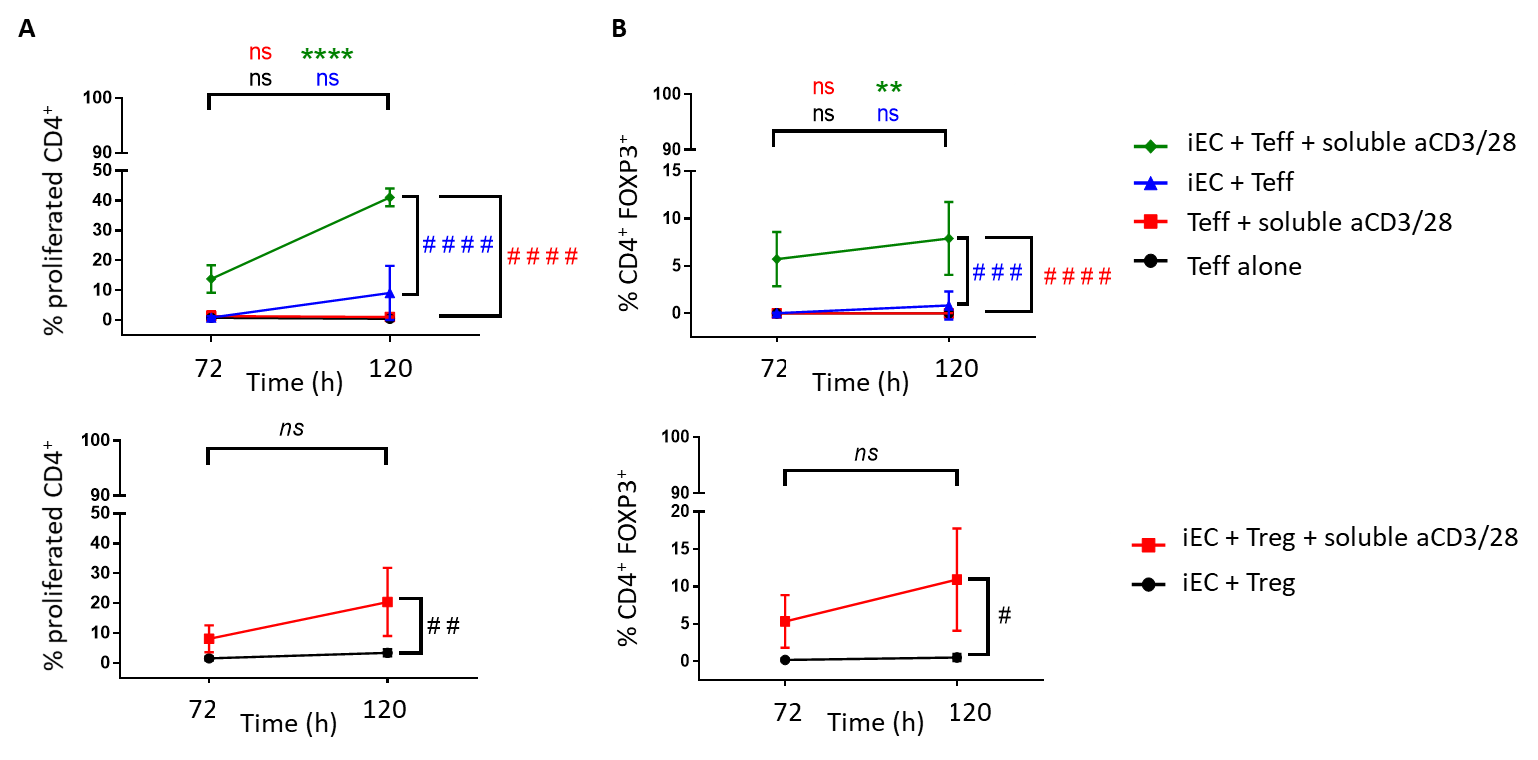
**

In order to study the effect of human EC interaction specifically on Teffs and Tregs, these subsets were isolated separately by cell sorting and cocultured separately with HUVECs. **(A)** When Teffs were cocultured with IFN-γ-stimulated HUVECs, significant levels of proliferation compared to non-cytokine stimulated HUVEC were seen with concurrent aCD3/28 stimulation. Similarly, when Tregs where cocultured with IFN-γ-stimulated HUVECs and aCD3/28, significant proliferation was observed compared to non-cytokine stimulated HUVEC cocultures. **(B)** The proliferated cells in Teff-HUVEC cocultures with aCD3/28 showed significantly increased FOXP3 expression levels by 72 h which was further increased by 120 h. This was similar for aCD3/28 stimulated Treg-HUVEC cocultures. Two-way ANOVA with Tukey test was conducted; ****P<0.0001, **P<0.01 120 h cf. 72 h data; ####P<0.0001, ###P<0.001, ##P<0.01, #P<0.05 for 120 h data cf. to Teff alone control or iEC + Treg alone control at 120 h; n=5.

**Figure C: Sorting of Teff and Tregs and purity of sorted populations**

**
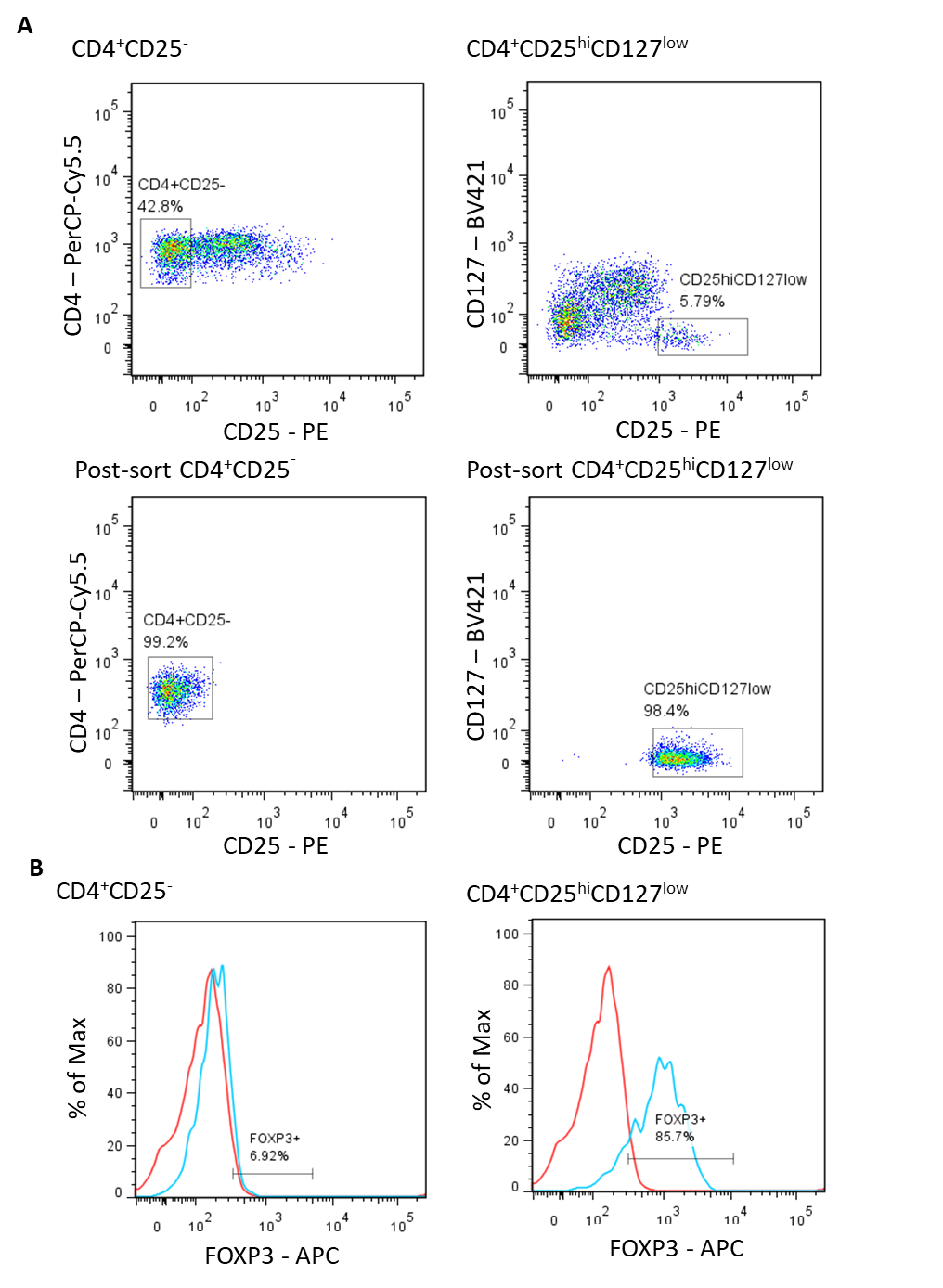
**

**(A)** CD4^+^CD25^-^ T cells were sorted as Teffs whereas CD4^+^CD25^hi^CD127^low^ T cells were sorted as Tregs, plots show that FACS yield populations of Teffs and Tregs of high purity. **(B)** FOXP3 expression in the CD4^+^CD25^-^ and CD4^+^CD25^hi^CD127^low^ cell populations was analysed by flow cytometry whereby percentage of FOXP3^+^ cells for each population was acquired. Data was collated from 29 different donors and showed that sorted CD4^+^CD25^-^ T cells had 6.96 ± 4.12 % FOXP3^+^ cells whereas 87.61 ± 6.74% of sorted CD4^+^CD25^hi^CD127^low^ T cells expressed FOXP3. Two-tailed paired T-test was used; P<0.0001, n=29.

**Figure D: Cytokine production profile of unstimulated and cytokine-activated HDMECs**


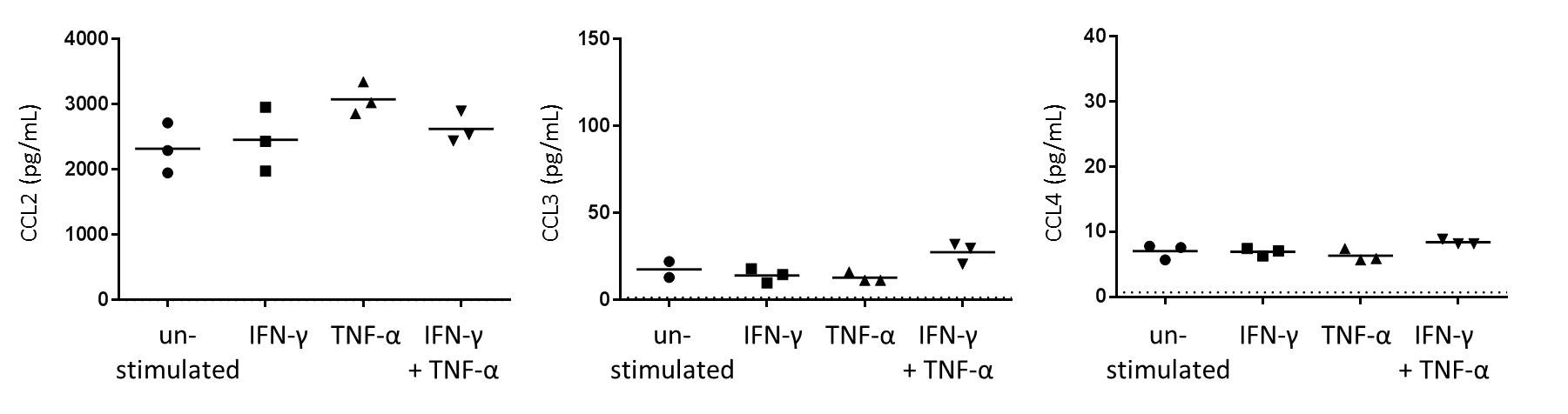


HDMECs were plated at 1 x 10^5^ cells/ well in 24-well plates were kept unstimulated of activated with 10 U/mL IFN-γ, 1 ng/mL TNF-α or a combination of both cytokines for 24 h. Cells were then washed and media replaced with 1 mL complete RPMI media. After 24 h, conditioned media was collected and cytokine measurements were completed using bead-based multiplex assays. Luminex® Bead sets (Bio-Rad) used included those for cytokines CCL2, CCL3, CCL4, FGF-basic, G-CSF, GM-CSF, IFN-γ, IL-1α, IL-1ra, IL-1β, IL-2, IL-4, IL-5, IL-6, IL-10, IL-17, TNF-α and VEGF. Only production of CCL-2, CCL-3, CCL-4, IL-6, G-CSF and GM-CSF were detected and CCL-2, CCL-3 and CCL-4 are shown in this figure with means drawn. Dotted lines indicate basal levels in RPMI media. One-way ANOVA with Dunnett’s test was conducted; n=3.

**Figure E: CD25 expression of CD4^+^ T cells in HUVEC cocultures**

**
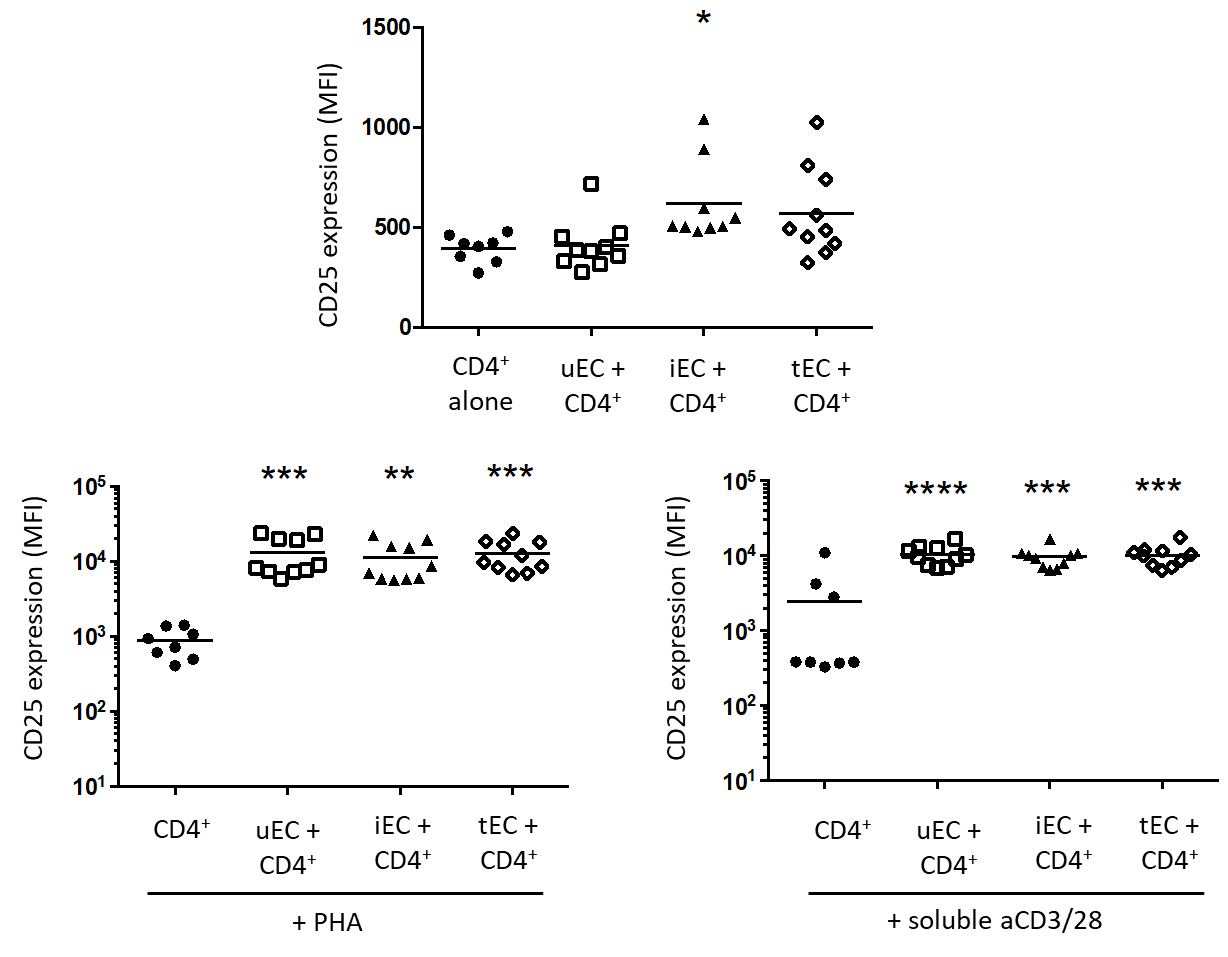
**

Cocultures of CD4^+^ T cells with unstimulated (uEC), IFN-γ- (iEC) or TNF-α-stimulated (tEC) HUVECs in the absence of external stimuli, or with addition of PHA or stimulatory aCD3/28 antibodies were conducted. Proliferated CD4^+^ T cell populations from these cocultures were analysed at 72 h for their CD25 expression levels. In CD4^+^ T cell-HUVEC alone cocultures, only proliferated CD4^+^ T cells from cocultures with IFN-γ-stimulated HUVEC showed increase in CD25 expression. When cocultures were stimulated with either PHA or aCD3/28 antibodies, proliferated CD4^+^ T cells showed significantly high levels of CD25 expression compared to CD4^+^ T cells alone cultures. One-way ANOVA with Tukey test was conducted; ****P<0.0001, ***P<0.001, **P<0.01 and *P<0.05 cf. control; n=10.

**Figure F: Production of nitric oxide and TGF-β by unstimulated and cytokine-activated HUVECs**
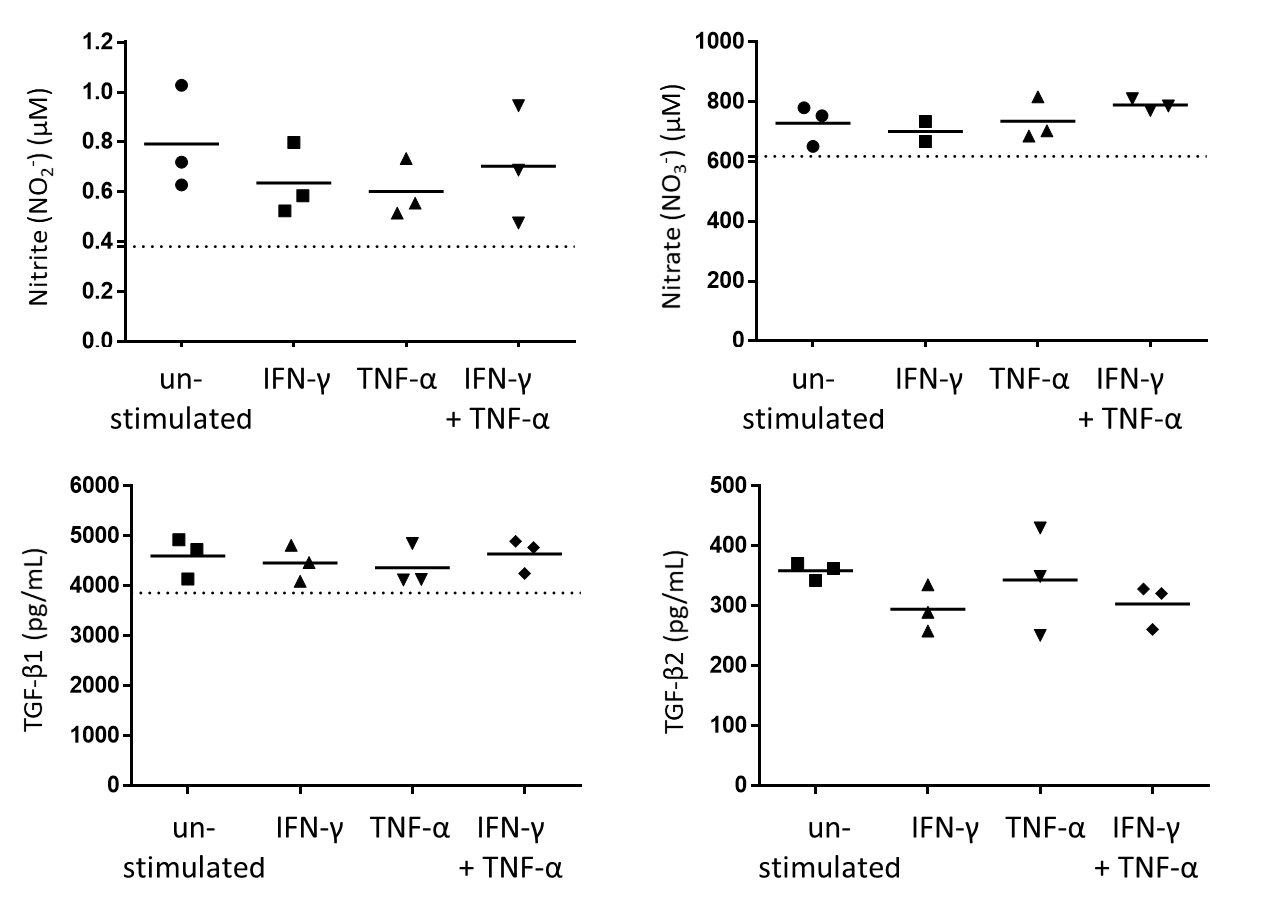


HUVECs were plated at 1 x 10^5^ cells/ well in 24-well plates were kept unstimulated of activated with 10 U/mL IFN-γ, 1 ng/mL TNF-α or a combination of both cytokines for 24 h. Cells were then washed and media replaced with 1 mL complete RPMI media. After 24 h, conditioned media was collected. For measurement of NO_2_^-^ and NO_3_^-^ levels, protein was removed from each sample by precipitation using methanol and then analysed using an ENO-20 NOx analyser. For measurement of TGF-β1 and TGF-β2 levels, ELISAs were performed using the Human TGF-beta 1 DuoSet® and Human TGF-beta 2 Duoset® ELISA development systems from R&D systems (for TGF-β1, samples were acid activated). Cytokine stimulation did not stimulate an increase in production of any of the soluble mediators described above into the HUVEC conditioned media. Dotted lines indicate basal levels in RPMI media. One-way ANOVA with Dunnett’s test was conducted; n=3.
